# The Effect of Adding Fentanyl to Epinephrine-Containing Lidocaine on the Anesthesia of Maxillary Teeth with Irreversible Pulpitis: A Randomized Clinical Trial

**Published:** 2014-10-07

**Authors:** Payman Mehrvarzfar, Anahita Pourhashemi, Fatemeh Khodaei, Behnam Bohlouli, Farzin Sarkarat, Mojdeh Kalantar Motamedi, Mohammad Karim Layegh Nejad, Sara Zamaheni

**Affiliations:** a *Department of Endodontics, Dental Branch, Islamic Azad University, Tehran, Iran;*; b *Department of Restorative Dentistry, Dental School, Shahid Beheshti University of Medical Science, Tehran, Iran;*; c *Private Practice, Tehran, Iran;*; d *Department of Oral and Maxillofacial Surgery, Dental Branch, Islamic Azad University, Tehran, Iran;*; e *Department of Endodontics, Dental School, Shahid Beheshti University of Medical Science, Tehran ,Iran*

**Keywords:** Fentanyl, Infiltration, Irreversible Pulpitis, Lidocaine, Local Anesthesia, Maxillary Molars

## Abstract

**Introduction:** Deep and long-lasting anesthesia is essential throughout endodontic treatment. This study was conducted to compare the effect of adding fentanyl to epinephrine-containing lidocaine on depth and duration of local anesthesia in painful maxillary molars with irreversible pulpitis (IRP). **Methods and Materials:** This randomized double-blind, clinical trial with parallel design was conducted on 61 healthy volunteers; the control group received a mixture of normal saline and 2% lidocaine with 1:80000 epinephrine and the experimental group received a mixture of fentanyl and 2% lidocaine with 1:80000 epinephrine. The depth and duration of pulpal anesthesia were evaluated by means of electric pulp testing in 5-min intervals during a period of 60 min. Pain intensity was recorded five times: before injection, after injection, during access cavity preparation, initial file placement and pulpectomy using visual analog scale (VAS). All data were analyzed and compared using the chi-square and Mann-Whitney tests. **Results: **Except for one patient in the control group, all others had deep and long-lasting anesthesia. The difference between pain intensity of the control and experimental groups was not statistically significant (*P*>0.05). **Conclusion: **Addition of fentanyl to conventional local anesthetic solution did not increase the effectiveness of infiltration in patients diagnosed with IRP.

## Introduction

Effective anesthesia is one of the most important requirements in endodontic practice. Although infiltration of 2% lidocaine with epinephrine is reported to be successful for maxillary teeth [1-3], achieving a deep and long-lasting anesthesia is not always easy especially in treating “hot” teeth with signs of symptomatic irreversible pulpitis (IRP) [4, 5].

It is found that almost half of patients diagnosed with IRP in maxillary teeth did not experience a pain-free endodontic treatment after buccal infiltration of 4% articaine with 1:100000 epinephrine or 2% lidocaine with 1:80000 epinephrine [1]. In another clinical trial, the success of maxillary infiltration with 2% lidocaine containing 1:200000 epinephrine in patients with IRP was reported to be 27%, where the success was defined as the absence of pain during access preparation and root canal instrumentation [6]. On the other hand, a recent study has shown that maxillary buccal infiltration did not provide adequate pulpal anesthesia during endodontic treatment of palatal root in maxillary first molars with IRP. In addition, the heart rate significantly increased in these patients during negotiation of palatal canals [7].

A number of reasons have been mentioned for incomplete anesthesia, such as low pH of inflamed tissues at the injection site and less conversion of the local anesthetic agents to their non-ionized fat-soluble form which is necessary to penetrate the nerve sheath [8, 9]. Another explanation for the failure is that nerves within the inflamed tissue have altered resting potentials and decreased excitability thresholds [10]. Another factor may be the increase in the number of TTX-R (tetrodotoxin resistant) sodium channels, in an inflamed dental pulp [11].

Several techniques have been suggested to solve problems related to the anesthesia of maxillary teeth, which include the use of various local anesthetic agents, increasing the volume of the local anesthetic agent and the application of supplementary injections [11]. However, a change in the type of the anesthetic agent may not be very effective, especially in teeth with IRP [5, 12]. In addition, problems such as higher toxicity of increased volume of the anesthetic agent and a need for repeated injections should be taken into consideration.

In recent years, combinations of low doses of analgesic opioids with conventional anesthetic solutions, such as lidocaine have been reported to improve the efficacy and duration of local anesthesia [9, 13]. Fentanyl is a fast-acting potent synthetic agonist of *μ* receptors in the central and peripheral nervous systems [9, 14]. Some researchers have claimed that fentanyl may increase the efficacy of commonly used local anesthetic agents, especially in teeth with symptomatic irreversible pulpitis, through a peripheral opioid action [9, 13]. Human studies have valued the role of local inflammation in peripheral opioid analgesic receptors [15, 16]. In addition, the presence of a large number of opioid receptors has been shown on the surface of C fibers in the pulp, which are responsible for the induction of pain [17]. Therefore, it may be possible that the pulpal pain in IRP may respond practically to the administration of peripheral opioids [9, 18]. Several studies on the intraligamentary injection of fentanyl combined with local anesthetic agents have mentioned promising results in enhancing the success of local anesthesia [9, 13]. However, some failed to show a significant difference between a combination of lidocaine and fentanyl with lidocaine containing epinephrine in maxillary infiltration injections in inflamed dentoalveolar tissues [14].

To date, no studies have evaluated the effect of adding fentanyl to epinephrine-containing lidocaine on efficacy of local anesthesia in maxillary teeth with symptomatic IRP using the infiltration technique. Moreover, there are some controversies regarding the efficacy of the local fentanyl in increasing the depth and duration of local anesthesia in inflamed tissues. Therefore, the present study was designed to compare the efficacy of 2% lidocaine with 1:80000 epinephrine with and without fentanyl, on the depth and duration of local anesthesia after infiltration injection in maxillary molars with symptomatic IRP.

## Methods and Materials

The study was approved by the Committee of Ethics at the Islamic Azad University, Dental Branch, Tehran, Iran (Grant no. 18050). This protocol was also registered at ClinicalTrials.gov (identification no.: NCT01794533). Sixty-one adult patients were assessed in this prospective randomized double-blind, clinical trial with parallel design. Participants were selected consecutively from patients referring to the Endodontic Department from October first to November 27^th^, 2012. An informed consent was signed by each volunteer. The sample size was calculated using the data of Rattan`s study (mean and standard deviations for plain: 19±4.37 in lidocaine and 15.3±3.62 for lidocaine+fentanyl, *α*=0.05 and *β*=0.2) and a possible rate of loss to follow-up of 30% [14].

***Inclusion criteria:***

Patients were aged between 18 and 65 years with no systemic diseases categorized as Class I ASA. Other inclusion criteria were: individuals requiring urgent root canal treatment of maxillary first or second molars; teeth with symptoms of IRP (moderate to severe spontaneous nocturnal pain) with a positive response to thermal vitality tests [a long painful response to a cold test with Endo-Ice (Hygienic Corp., Akron, OH, USA), lasting for at least 15 sec] and no clinical or radiographic signs or symptoms of acute or chronic apical periodontitis; and no history of analgesic consumption 12 h prior to assessment.

***Exclusion criteria:***

Any systemic condition that could potentially alter the treatment protocol, allergic reaction to opioids, benzodiazepines, barbiturates, pregnancy and lactation, contraindication of the use of epinephrine (such as unstable angina), and non-vital pulp after access cavity preparation, led to exclusion of the patient from the study.

Thorough medical and dental histories were taken from all patients. After extra oral and intraoral examination, diagnostic tests and radiographic assessment for absence of periradicular radiolucencies or advanced periodontal diseases, the clinical diagnosis of a symptomatic IRP was confirmed.

The objective electric pulp testing (EPT) and the subjective visual analog scale (VAS) were used for the evaluation and determination of depth and duration of anesthesia before starting treatment.

Before infiltration of the anesthetic agent, the tooth was evaluated twice by an EPT device (Analytic Technology, Redmond, WA, USA) and the responses were recorded; then a VAS ruler with a 0-170 scale was used to record pain intensity as follows: absence of pain (score 0), mild pain (score 0-54), moderate pain (score 55-114) or severe excruciating pain (score>114). Only patients with VAS score over 55 were included in the study. The subjects were assigned to the test and control groups using sequential randomized sampling technique and each subject received a random code number generated by computer algorithm to be placed in one of the two groups: The control group (*n*=30); local infiltration of 1.8 mL of 2% lidocaine containing 1:80000 epinephrine (Darupakhsh, Tehran, Iran) along with 0.8 mL of 0.9% sterile normal saline solution and the test group (*n*=31); local infiltration of 1.8 mL of 2% lidocaine, containing 1:80000 epinephrine and 0.8 mL of fentanyl (40 µg).

In this double-blind study the solutions were prepared by one operator who aseptically transferred the anesthetic agents into disposable 5-mL plastic syringes with a 27-guage needle for infiltration and the other operator injected the anesthetic agents. Each syringe had a randoml-coded label so that the second operator was blind to the content of the syringe and the patient was not aware of the type of tested drug. The code of each syringe used was recorded in each patient’s file.

**Table 1:** Demographic and baseline characteristics of each group

**Baseline characteristics**		**Control group**	**Experimental group**
**Age (year)**		34 (5)	31.5 (8)
**Gender**	**Female**	18 (60%)	19 (62%)
	**Male**	12 (40%)	12 (38%)
**Tooth type**		Maxillary molars	Maxillary molars
**Initial preoperative pain (VAS)**		111.166 (45.137)	113.333 (38.64)

As the topical anesthesia, a 20% benzocaine gel (Patterson Dental Supply, St Paul, MN, USA) was applied on the injection site for 30 sec. Then the anesthetic agent was injected supraperiosteally at the depth of the mucobuccal fold between the mesiobuccal and distobuccal roots adjacent to the apex of the target molar within 60 sec. All the injections were carried out by the same clinician.

The depth and duration of pulpal anesthesia were determined with EPT device [5, 19, 20] and VAS [14, 21, 22]. Two consecutive negative reading of EPT (maximum 80) immediately before preparing the access cavity and negative or mild pain during access cavity preparation, insertion of initial file and pulpectomy were considered as signs of successful anesthesia [5, 19, 20]. Likewise, failure was defined as the absence of any of the aforementioned criteria [22]. The tip of the EPT was placed on the midbuccal aspect of each tooth midway between the gingival margin and the occlusal surface after its isolation. A small amount of toothpaste was used as an electrolyte during each test. Also the depth of anesthesia was determined using VAS, one minute after injection, during access cavity preparation, initial file placement and finally pulpectomy. Success of an effective anesthetic procedure was defined based on the absence of pain or presence of mild pain (VAS scores 0-54) during different stages of root canal treatment [9, 20, 21].

All data were analyzed by the chi-square test for EPT findings and the Mann-Whitney test for VAS readings. Statistical significance was set at 0.05.

## Results

A total of 61 patients were evaluated; there were no significant differences between the two test and control groups in terms of patients’ age, gender and pain severity at baseline (*P*>0.05). Regarding the depth and duration of pulpal anesthesia based on EPT results, 29 subjects (96.6%) in the control and all the subjects (100%) in the test groups exhibited successful pulpal anesthesia, with no statistically significant difference within 60 min (*P*>0.05).

Table 1 presents the success rates of pulpal anesthesia at different stages of root canal treatment based on VAS and no significant differences were observed between the control and test groups (*P*>0.05).

## Discussion

Unfortunately, infiltration or nerve-blocking injection techniques are not always successful in teeth with painful symptomatic IRP which by itself leads to patient and operator’ stressful root canal treatment [4]. Nociceptors (C fibers) are the main nerve fibers responsible for induction of pain in inflammatory tissue reactions. On the other hand presence of a large number of opioid receptors has been shown on the surface of C fibers in the pulp [17]. Furthermore, during inflammatory conditions, there is an increase in axonal transport of opioid receptors from the dorsal root ganglia toward the peripheral nerve endings, resulting in an increase in the number of *μ* opioid receptors in the inflamed peripheral tissues [14, 23]. Opioid drugs combined with local anesthetics have been successful in inducing effective epidural and spinal cord anesthesia and may function synergistically at the spinal cord level in combination with local anesthetic agents [24, 25].

Opioids have a dual mechanism of action [26, 27]; the primary effect is related to their interaction with opiate receptors in the spinal cord. However, at high concentrations they may also reduce the action potential of A and C fibers similar to the phenomenon with local anesthetics. Also, a recent study indicated that fentanyl decreases the discharges of C and A nociceptors to higher levels than the threshold in chronic inflammation [28]. Considering this, the hypothesis of the present study was that activating *μ* opioid peripheral receptors through adding fentanyl, can potentially lead to possible reduction in transmission of pain signals from the pulpal nerves toward the central nervous system (CNS). However, no significant effects were observed with a combination of 40 μg of fentanyl and 2% lidocaine with 1:80000 epinephrine. In fact, in both test and control groups, there was complete success in achieving maxillary infiltration anesthesia.

In previous studies on the evaluation of maxillary infiltration technique using an EPT, a success rate of 64-100% has been reported [22]. However, it is difficult to compare the results of the present study with those of previous ones due to the differences in methodologies including tooth type, baseline inflammatory condition of the pulp, and the type and amount of the local anesthetic agent tested. 

Our present findings are to some extent in agreement with the results of a study conducted by Rattan *et al.* [14], who reported that incorporation of 40 μg of fentanyl to 2% lidocaine did not result in an increase in the quality of anesthesia during surgical extraction of teeth in inflamed dentoalveolar tissues. However, in an earlier study, a combination of morphine and articaine resulted in an improvement in the efficacy of inferior alveolar nerve block in surgical models of inflamed teeth [16]. In this context, different injection sites (maxilla or mandible), the type of injection, the number of samples and the methodology should be taken into consideration. Problems with the use of morphine combined with local anesthetic agents in peripheral tissues include the low effect rate (15-20 min), increase in histamine release at the injection site and local inflammation that may aggravate the pain severity [14]. Fentanyl is a synthetic phenylpiperidine, which is specific to *μ* receptors; in addition to high analgesic effect and rapid onset of effect, it causes milder disturbances in hemodynamic balance and does not induce the release of histamine [14].

**Table 2:** Intensity of pain recorded in five treatment stages using VAS based on treatment groups

**Treatment stages**	**Groups**	**Pain intensity**	***P-*** **value**
**After injection**	Control group	64.833 (1.532)	0.6
	Experimental group	56.666 (2.841)	
**Access cavity preparation**	Control group	1.333 (7.302)	0.3
	Experimental group	0	
**Initial file placement**	Control group	1.666 (9.128)	0.3
	Experimental group	0	
**Pulpectomy**	Control group	8.166 (17.144)	0.2
	Experimental group	5.333 (14.31)	

In our study a 40-μg safe dose of fentanyl was selected with proper diffusion in body tissues but because this dose is less than 50 μg, it had no systemic effects on our participants [15]. 

In a previous study, the effect of supplementary intraligamentary injection of peripheral opioids combined with 2% mepivacaine, containing 1:200000 epinephrine, was assessed during root canal treatment of maxillary first molars with symptomatic IRP [9]. The results showed that incorporation of a very small dose of fentanyl (0.4 mL of 0.05 mg/mL fentanyl) resulted in a significant increase in depth of anesthesia and decreased the pain after supplementary intraligamentary injection subsequent to a standard maxillary infiltration anesthetic procedure with 1.8 mL of 2% mepivacaine containing 1:200000 epinephrine. Although the mentioned study was very similar to the present investigation in terms of sample selection, the different injection technique and the type of the tested anesthetic agent might explain this disagreement. On the other hand, some studies have failed to show that combination of opioids with local anesthetic agents is effective for inferior alveolar nerve block [21]. The failure of the synergistic function of fentanyl and lidocaine may be attributed to differences in the physical and chemical properties of these two drugs [14]. The peripheral analgesic action of opioids using different opioids and dosages along with various local anesthetics need to be evaluated further in future studies.

In this study the primary contributing factors, such as the initial inflammatory condition of the pulp and pain severity in all of the samples were matched; two subjective and objective techniques were used simultaneously in order to evaluate the success rate of pulpal anesthesia. In the present study a maximum EPT reading of 80 was used to determine the depth and duration of pulpal anesthesia [29, 30]. The absence of patient response to two consecutive negative EPT readings of 80 is a reliable technique to determine the depth of pulpal anesthesia in vital asymptomatic teeth. However, in teeth with symptomatic IRP this technique alone may not be able to suitably evaluate the depth of pulpal anesthesia. Therefore, VAS was also used during the procedures to determine the severity of pain (depth of anesthesia) in order to achieve more reliable clinical results. Some of the drawbacks of such human studies include an inability to match all the patients’ related factors such as pain threshold, the extent of pulpal inflammation and also different host capacity to metabolize tested medications.

Although, a combination of fentanyl and lidocaine exhibits a clear synergistic effect at the spinal cord level or when used as an intraligamentary injection, it may not be possible to significantly achieve this effect in maxillary infiltration techniques. It is recommended that lower doses of anesthetic solutions combined with various opioid drugs be evaluated.

## Conclusion

In summary, the success of maxillary infiltration technique with 2% lidocaine with 1:80000 epinephrine was significantly high in maxillary molars with symptomatic irreversible pulpitis. However, a combination of fentanyl and lidocaine did not increase the effectiveness of maxillary infiltration technique.
